# Staurosporines decrease ORMDL proteins and enhance sphingomyelin synthesis resulting in depletion of plasmalemmal phosphatidylserine

**DOI:** 10.1038/srep35762

**Published:** 2016-11-02

**Authors:** Masashi Maekawa, Minhyoung Lee, Kuiru Wei, Neale D. Ridgway, Gregory D. Fairn

**Affiliations:** 1 Keenan Research Centre for Biomedical Science, St. Michael’s Hospital, Toronto, ON, Canada; 2 Department of Biochemistry, University of Toronto, Toronto, ON, Canada; 3 Departments of Pediatrics, and Biochemistry & Molecular Biology, Atlantic Research Centre, Dalhousie University, Halifax, Nova Scotia, Canada; 4 Department of Surgery, University of Toronto, Toronto, ON, Canada

## Abstract

Accumulation of phosphatidylserine in the inner leaflet of the plasma membrane is a hallmark of eukaryotes. Sublethal levels of staurosporine and related compounds deplete phosphatidylserine from the plasma membrane and abrogate K-Ras signaling. Here, we report that low-dose staurosporine and related compounds increase sphingomyelin mass. Mass-spectrometry and metabolic tracer analysis revealed an increase in both the levels and rate of synthesis of sphingomyelin in response to sublethal staurosporine. Mechanistically, it was determined that the abundance of the ORMDL proteins, which negatively regulate serine-palmitoyltransferase, are decreased by low-dose staurosporine. Finally, inhibition of ceramide synthesis, and thus sphingomyelin, prevented the displacement of phosphatidylserine and cholesterol from the inner leaflet of the plasma membrane. The results establish that an optimal level of sphingomyelin is required to maintain the distribution of phosphatidylserine and cholesterol in the plasma membrane and further demonstrate a complex relationship between the trafficking of phosphatidylserine and sphingomyelin.

The plasma membrane provides many essential functions for cell viability but acts primarily as a protective barrier and a conduit for entry. By its nature, the plasma membrane takes part in many physiologically important events such as endocytosis, exocytosis, ion and metabolite exchange, signal transduction, and cell-cell interaction. Like all biological membranes, the plasma membrane in metazoans is a phospholipid bilayer that contains cholesterol and membrane proteins^1^. However, the plasma membrane has a unique asymmetric distribution of phospholipids and sphingolipids that is essential for its biological activity^2^,^3^. For instance, phosphatidylserine (PtdSer) is maintained in the cytosolic leaflet while sphingomyelin (SM) is found in the exofacial leaflet. PtdSer and SM are unique and have been described to associate with cholesterol. Additionally, the importance of PtdSer in the inner leaflet of the PM is further magnified by the reported asymmetric distribution of cholesterol, which is ostensibly more abundant in the inner leaflet of the plasma membrane^4^. The topology of plasmalemmal lipids is regulated by a variety of mechanisms including; (a) phospholipid transporters (e. g. flippases, floppases, scramblases), (b) the membrane topology of the sphingolipid-synthesizing enzymes and (c) spontaneous flip-flop in the case of cholesterol^5^,^6^,^7^,^8^.

PtdSer in the cytosolic leaflet of the plasma membrane supports a variety of cellular functions by contributing to negative surface charge and retaining cholesterol both of which are important to support Ras nanocluster formation^9^,^10^,^11^,^12^. While in select stimulated or apoptotic cells, PtdSer is exported to the exofacial surface where it serves as a platform for blood coagulation or acts as an “eat-me” signal for phagocytosis^13^,^14^. To date, the mechanisms involved in the accumulation and retention of PtdSer in the cytosolic leaflet of the plasma membrane remains incompletely understood^3^. A recent study suggests that select oxysterol binding protein-related proteins (ORPs) transfer PtdSer directly from the endoplasmic reticulum (ER) to the plasma membrane in yeast^15^. In yeast and mammalian cells, phosphatidylinositol 4-phosphate (PI4P) has been proposed to drive counter-transport of PtdSer from the ER to the plasma membrane by specific ORPs^16^,^17^. Alternatively, vesicular transport of PtdSer from intracellular compartments, such as the Golgi apparatus and endosomes, to the plasma membrane will also contribute to the plasma membrane pool^18^. Indeed, inhibition of exocytosis in yeast results in the mislocalization and loss of polarization of PtdSer in the plasma membrane^19^.

Recently, a high-throughput chemical screen identified staurosporine (STS) and related compounds as potent disruptors of the plasmalemmal localization of K-Ras and PtdSer in Madin-Darby canine kidney (MDCK) cells independent of their ability to inhibit protein kinase C^20^. Treatment of cells with sublethal concentrations of STS caused a PtdSer biosensor^9^ to relocalize from the plasma membrane to intracellular compartments including recycling endosomes in MDCK cells^20^. This result suggests that STS impairs the endosomal sorting and recycling of PtdSer^20^, although the molecular mechanism of this relocalization and the impaired retrieval of PtdSer to the PM remain unclear.

In this study, we found that Chinese hamster ovary (CHO) cells had increased levels of SM after treatment with low dose STS. This elevation in SM mass accompanies the displacement of PtdSer from the plasma membrane. Lipidomic analysis showed that all the major SM species in CHO cells were increased upon treatment with STS or an analog. Additionally, radiolabelling with [^3^H]serine revealed enhanced incorporation into SM, but not other lipids, in STS-treated CHO cells. Further analysis determined that the abundance of the ORMDL proteins, negative regulators of serine-palmitoyltransferase reaction^21^, are reduced upon treatment with low-dose STS. Importantly, perturbation of PtdSer distribution was prevented by inhibition of SM synthesis, indicating a highly coordinated mechanism to maintain the PM composition that is dependent on SM synthesis and transport.

## Results

### Redistribution of phosphatidylserine and cholesterol in staurosporine-treated cells

**Figure 1:**
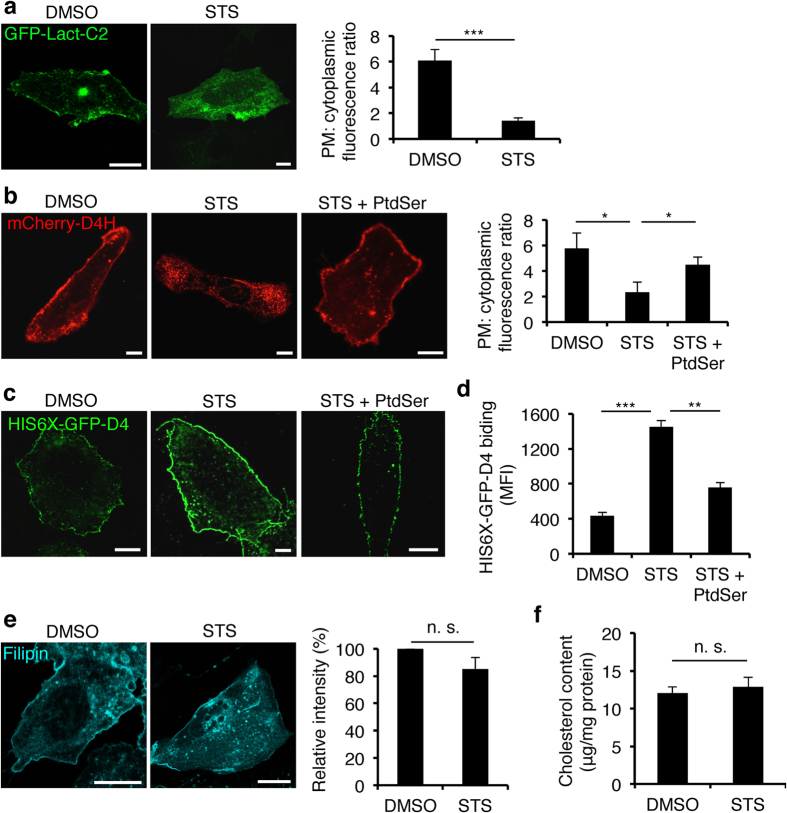
Distribution of PtdSer and cholesterol in the plasma membrane of staurosporine-treated CHO cells. (**a**) Confocal images of DMSO or staurosporine (STS) treated CHO cells expressing the PtdSer probe, GFP-Lact-C2. Bar, 10 μm. As described in the Methods section, the enrichment of fluorescence signal in the plasma membrane compared to the cytoplasm is shown (plasma membrane:cytoplasm) for GFP-Lact-C2 quantified from 50 cells from three independent experiments. Data are means ± SEM. ***p < 0.001. (**b**) Confocal images of DMSO, STS or STS + PtdSer treated CHO cells expressing mCherry-D4H. Bar, 10 μm. Ratio of fluorescence signal for mCherry-D4H determined as for the GFP-Lact-C2. (**c**) Confocal images of CHO cells treated with DMSO, STS or STS plus supplemented with exogenous PtdSer were labeled with His-GFP-D4 protein to detect exofacial leaflet cholesterol. Bar, 10 μm. (**d**) Binding of the His-GFP-D4 protein to the exofacial leaflets of the plasma membrane in DMSO, STS or STS + PtdSer treated CHO cells. Data are means ± SEM (n = 3). ***p < 0.001; **p < 0.01. MFI; mean fluorescence intensity. (**e**) Confocal images of DMSO or STS treated CHO cells stained with filipin to visualize the cellular distribution of cholesterol. Bar, 10 μm. Total cellular fluorescence intensity of filipin was quantified from 50 cells from three independent experiments. Data are means ± SEM. n. s., not significant. (**f**) The content of cholesterol in STS-CHO cells. Cells were collected from a 10 cm dish and the cholesterol content of the cells was then measured using a cholesterol oxidase-based Amplex Red kit. Data are means ± SEM (n = 3). n. s., not significant.

Previous studies have demonstrated that sublethal concentrations of staurosporine (STS) disrupt plasmalemmal localization of PtdSer in MDCK cells^20^ and CHO cells^11^. GFP-Lact-C2, a phosphatidylserine (PtdSer) biosensor^9^, dissociated from the plasma membrane and relocated to the intracellular compartments in STS-treated CHO (STS-CHO) cells (Fig. 1a). Consistent with our previous findings, in STS-CHO cells, mCherry-D4H, a biosensor for cholesterol in the cytosolic leaflets of the cellular membrane^11^, also dissociated from the plasma membrane (Fig. 1b). Supplementation with exogenous PtdSer caused mCherry-D4H to relocalize to the cytosolic leaflet of the plasma membrane consistent with the re-acquisition of cholesterol (Fig. 1b). Conversely, cholesterol accessibility in the exofacial leaflet of the plasma membrane was increased upon treatment with STS, and supplementation with PtdSer restored this phenotype in STS-CHO cells^11^ (Fig. 1c,d). The altered distribution of cholesterol did not alter the content of unesterified cholesterol in total cell lysates (Fig. 1e,f) indicating that STS does not significantly alter cholesterol uptake and metabolism. Collectively, these data showed that, in STS-CHO cells, plasmalemmal cholesterol is preferentially accumulated in the exofacial leaflet of the plasma membrane with more cholesterol detectable in the cytosolic leaflet of organelles.

### Increased plasmalemmal sphingomyelin in staurosporine-treated cells

**Figure 2:**
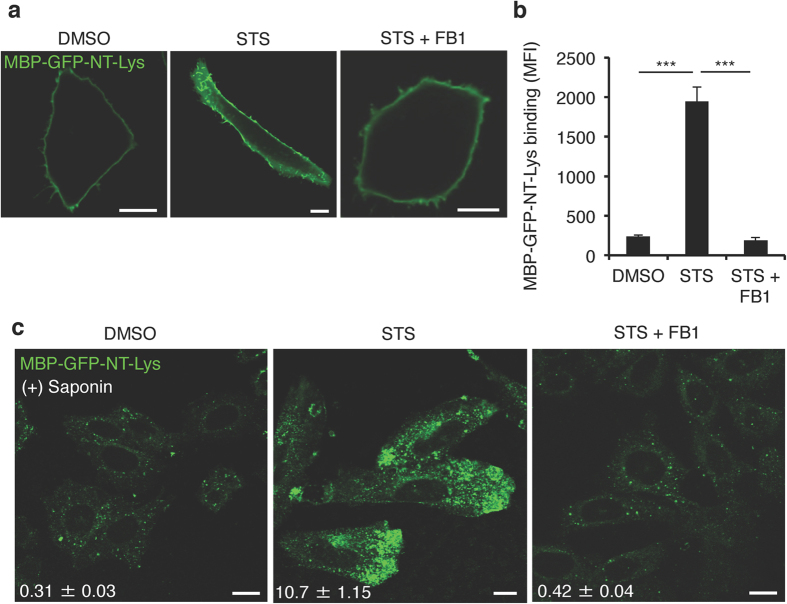
Staurosporine increases sphingomyelin contents in both the exofacial leaflet of the plasma membrane and intracellular compartments. (**a**) Confocal images of CHO cells treated with DMSO, 50 nM STS or 50 nM STS + 15 μM fumonisin B1 (FB1) for 24 hr labelled with MBP-GFP-NT-Lysenin (Lys) protein. Bar, 10 μm. (**b**) Binding of MBP-GFP-NT-Lys protein to the exofacial leaflets of the plasma membrane in DMSO, STS or STS + FB1 treated CHO cells. Data are means ± SEM (n = 3). ***p < 0.001. MFI; mean fluorescence intensity. (**c**) Confocal images of DMSO, STS or STS + FB1 treated CHO cells stained with MBP-GFP-NT-Lys protein after membrane permeabilization by 0.05% (w/v) saponin. Bar, 10 μm. The value of fluorescence intensity of MBP-GFP-NT-Lys was shown in images. Data are means ± SEM (50 cells from three independent experiments).

**Figure 3:**
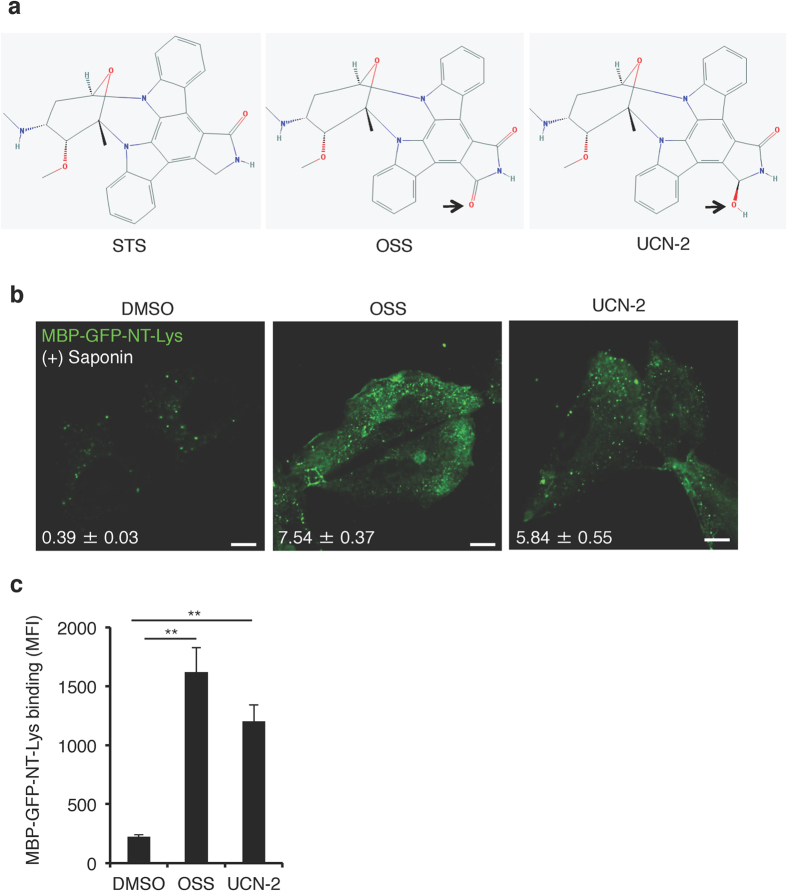
Staurosporine analogues also increase GFP-lysenin binding. (**a**) The molecular structure of STS, 7-oxostaurosporine (OSS) and UCN-2 obtained from PubChem (https://pubchem.ncbi.nlm.nih.gov/). Arrows indicate the differences in the structures of OSS and UCN-2 compared to STS. (**b**) Confocal images DMSO, 50 nM OSS or 100 nM UCN-2 treated CHO cells stained with MBP-GFP-NT-Lys protein after membrane permeabilization by 0.05% (w/v) saponin. Bar, 10 μm. The mean fluorescence intensity per pixel of MBP-GFP-NT-Lys was shown in images. Data are means ± SEM (50 cells from three independent experiments). (**c**) Binding of MBP-GFP-NT-Lys protein to the exofacial leaflet of the plasma membrane of CHO cells treated with DMSO, 50 nM OSS or 100 nM UCN-2 for 24 hr; Data are means values ± SEM (n = 3). **p < 0.01. MFI; mean fluorescence intensity.

To identify additional alterations in the PM of STS-treated CHO cells, we examined the abundance and subcellular distribution of sphingomyelin (SM), one of the predominant lipids found in the exofacial leaflet of the PM^7^,^22^. Due to its long saturated fatty acyl chain and large headgroup, SM selectively associates with and shields cholesterol from water molecules in membranes^23^. This characteristic of SM was postulated to enable it to form membrane nanodomains with cholesterol, so-called lipid rafts, in the exofacial leaflets of the plasma membrane^24^. We were interested in SM distribution in the plasma membrane in STS-CHO cells due to the increase in exofacial cholesterol (Fig. 1c,d). To visualize SM in the exofacial leaflet of the PM a non-toxic recombinant fragment of lysenin tagged with GFP (GFP-NT-Lys) protein^25^,^26^ was incubated with the cells. As illustrated in Fig. 2a,b, the binding of GFP-NT-Lys was significantly increased in STS-CHO cells. Additionally, staining of intracellular SM with GFP-NT-Lys following membrane permeabilization was also increased (Fig. 2c). Consistent with previous findings, treatment of CHO cells with two staurosporine homologs, 7-oxostaurosporine (OSS) and UCN-2, also altered PtdSer localization^20^ (Fig. 3a). Indeed, we confirmed that treatment of CHO cells with sub-lethal concentrations of OSS or UCN-2 also enhanced binding of GFP-NT-Lys to the intracellular membranes (Fig. 3b,c). Together, these results suggest that the staurosporine-family of compounds displace PtdSer from the PM and increase the levels or the accessibility of SM to lysenin.

### Lipidomics analysis reveals increased sphingomyelin abundance

**Figure 4:**
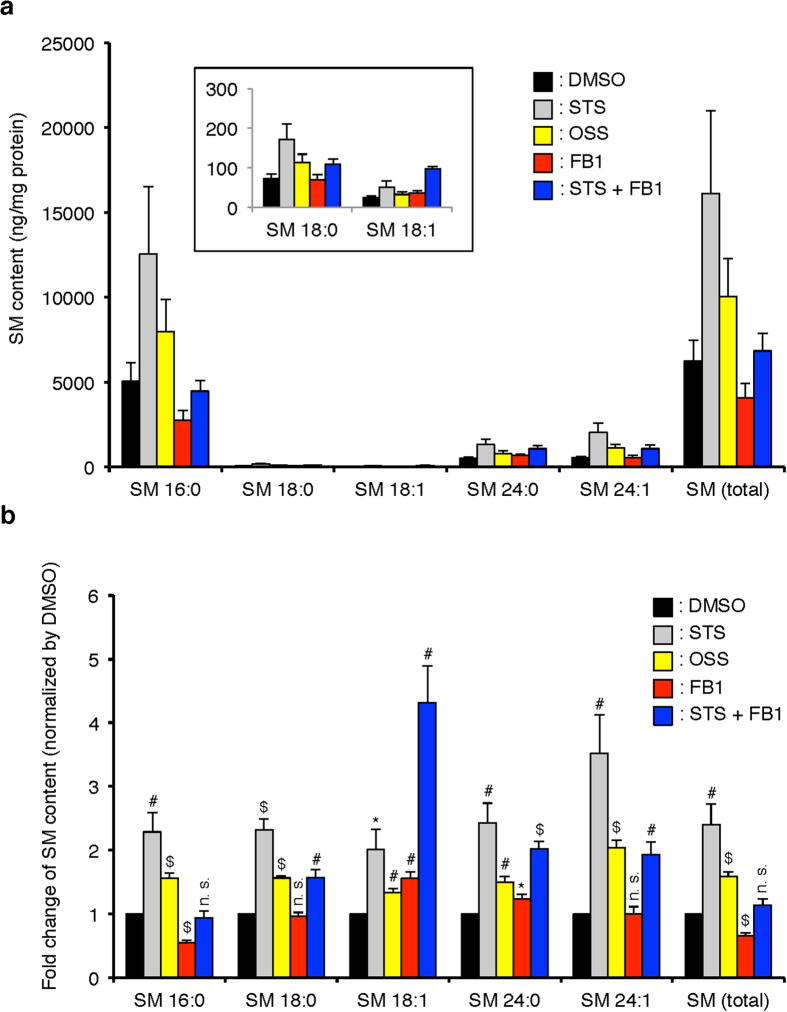
Determination of the molecular species of sphingomyelin following low-dose staurosporine. Quantitative lipidomic analysis of sphingomyelin species from control CHO cells and treated with 50 nM staurosporine (STS), 50 nM 7-oxostaurosporine (OSS), 15 μM fumonisin B1 (FB1) and 50 nM STS + 15 μM FB1 for 24 hours. Lipid composition is shown normalized to protein to compare the changes due to incubation with the drugs (**a**) and then data are normalized by the value of DMSO samples (**b**). Data are means ± SEM (n = 4). *p < 0.05; ^#^p < 0.01; ^$^p < 0.001; n. s., not significant vs. DMSO.

The lysenin protein engages 5 or 6 SM molecules at a time^27^. Thus, it was unclear if STS was changing the absolute amount of SM or its distribution within the membrane. Using quantitative mass spectrometry we found that STS and OSS treatment of CHO cells increase all molecular species of SM (Fig. 4a) while having minimal impact on the ceramide species (Supplemental Fig. 1). STS and OSS are known protein kinase C (PKC) inhibitors with comparable potency^28^. However, the effects on PtdSer and SM are likely independent of kinase inhibition at the concentrations used here, 10- to 20-fold lower than that used to inhibit PKC and induce apoptosis^20^. Thus, the structure-activity relation between PtdSer mislocalization and increased SM is likely independent of protein kinase C inhibition.

### Low-dose staurosporine enhances sphingomyelin biosynthesis

**Figure 5:**
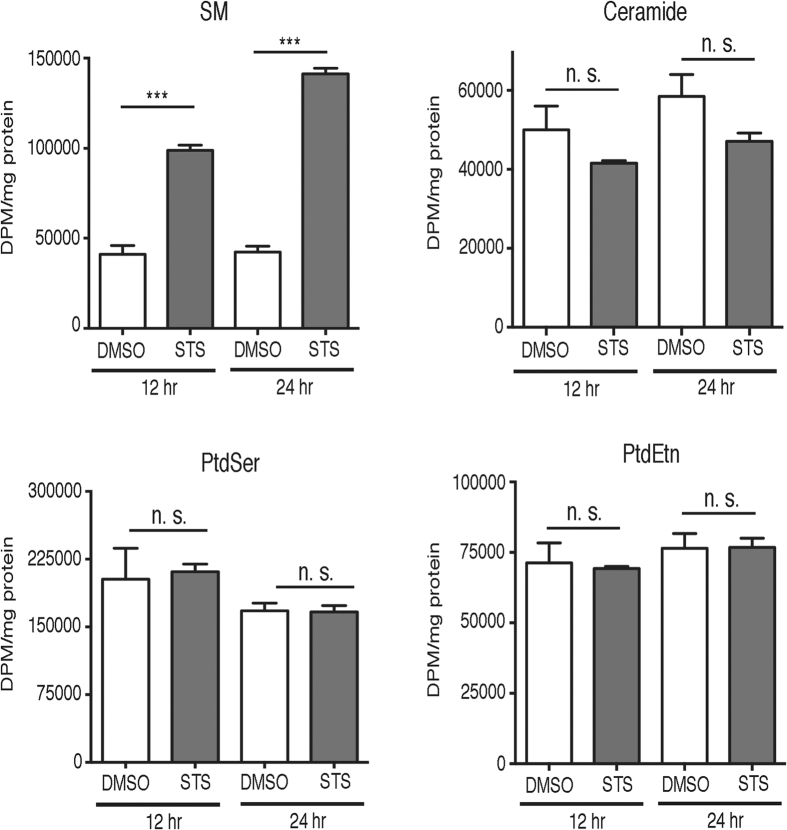
Staurosporine enhances the biosynthesis of sphingomyelin. CHO cells were treated with 50 nM STS for 12 or 24 hr. During the final 2 hr of each treatment, cells were pulse-labelled with [^3^H]serine and incorporation into PtdSer, PtdEtn and sphingolipids were quantified and normalized to total cellular protein. Results are the mean and standard error of 4 determinations. ***p < 0.001; n. s., not significant.

Increased SM mass caused by STS treatment could be the result of increased *de novo* synthesis pathway or inhibition of degradation. To distinguish between these two possible mechanisms, we performed [^3^H]serine labeling experiments as previously described^29^. The initial and rate-limiting step in SM biosynthesis catalyzes the condensation of L-serine and palmitoyl-CoA to produce 3-ketosphinganine^30^. Thus, pulse-labelling of cells with the precursor [^3^H]-serine is an accurate method to measure ceramide and SM synthesis. The incorporation of [^3^H]-serine into SM was significantly increased 2-3-fold by treatment with 50 nM STS for 12 or 24 hr compared to control cells (Fig. 5). Isotope incorporation into the intermediate ceramide was not significantly affected nor was the synthesis of PtdSer or its metabolic product phosphatidylethanolamine (PtdEtn) (Fig. 5). Together, these findings indicate that low-dose STS stimulates the *de novo* synthesis and accumulation of SM.

### Staurosporine decreases the abundance of ORMDL proteins

**Figure 6:**
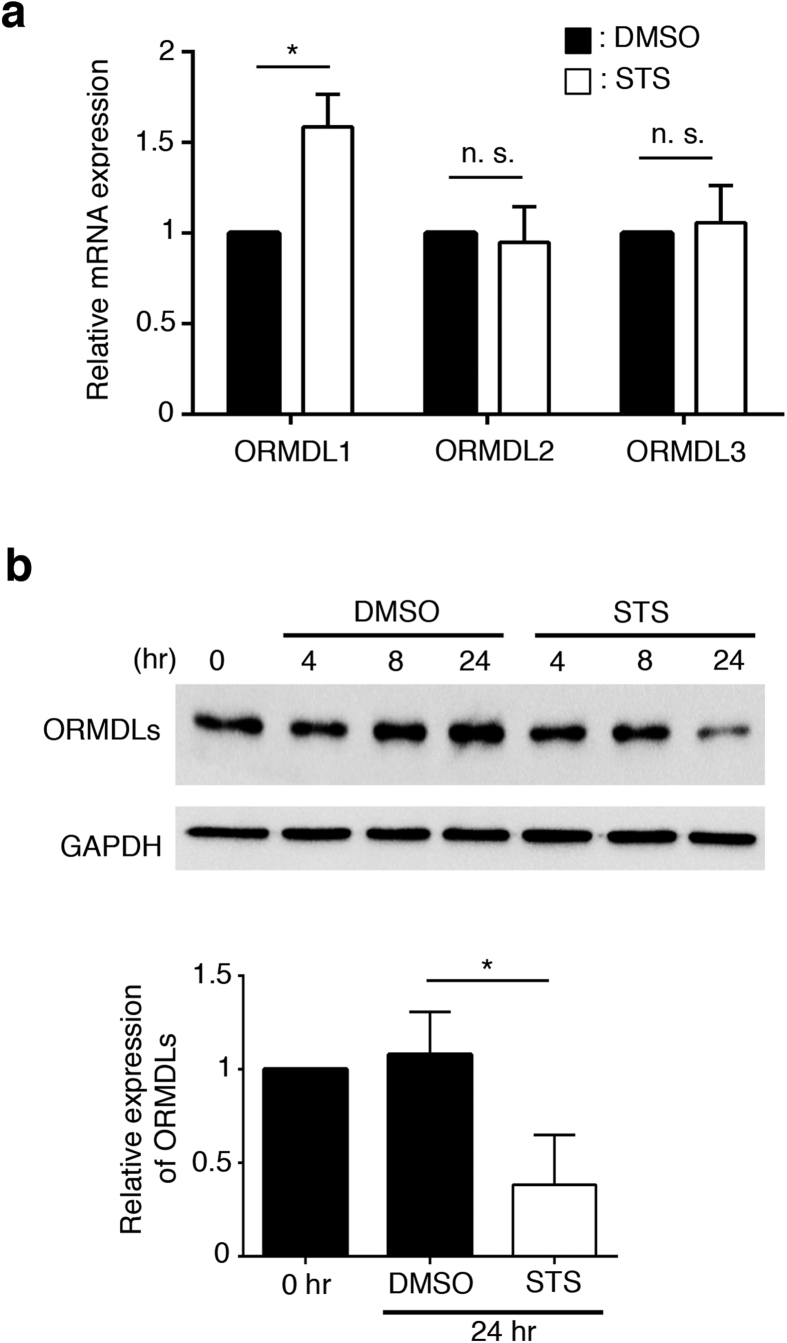
Staurosporine decreases the abundance of ORMDL proteins. (**a**) The expression level of ORMDLs mRNA in STS-treated CHO cells. CHO cells were treated with 50 nM STS for 24 hr and the amount of ORMDLs mRNA was measured by quantitative RT-PCR. Data were normalized to GAPDH mRNA and shown normalized by the value of DMSO-treated CHO cells. Data are mean values ± SEM (n = 3). *p < 0.05; n. s., not significant. (**b**) The expression level of the ORMDL proteins in STS-treated CHO cells. CHO cells were treated with 50 nM STS for 4, 8 or 24 hr and cell lysate were collected. Relative expression of the ORMDLs was quantified and normalized to GAPDH expression, and the data were normalized by the value of non-treated cells samples (0 hr). Data are means ± SEM (n = 3). *p < 0.05. Only the portions of the blot with relevant information is depicted. The full blot is included in the supplemental information.

The rate-limiting step in sphingolipid biosynthesis is catalyzed by the serine palmitoyltransferase complex^31^. First discovered in yeast and recently extended to mammalian cells, the Orm1-like (ORMDL) family of proteins act as ceramide sensors and feedback inhibitors of the SPT complex^21^,^32^. We hypothesized that STS-mediated stimulation of SM synthesis might occur via impaired expression of the ORMDL proteins. Using quantitative RT-PCR we found that low-dose STS had little impact on the levels of mRNA for ORMDL2 and ORMDL3 and caused a slight elevation in ORMDL1 mRNA (Fig. 6a). Next, using an antibody predicted to react with all three ORMDL proteins, we found that the abundance of the ORMDL proteins decreased ≈60% compared to control cells after a 24-hour incubation with 50 nM STS (Fig. 6b). Together these results indicate that STS treatment decreases the abundance of ORMDL at a post-transcriptional level and that removal of the feedback sensor is sufficient to enhance SM synthesis consistent with previous findings.

### Increased sphingomyelin synthesis is required to displace phosphatidylserine

**Figure 7:**
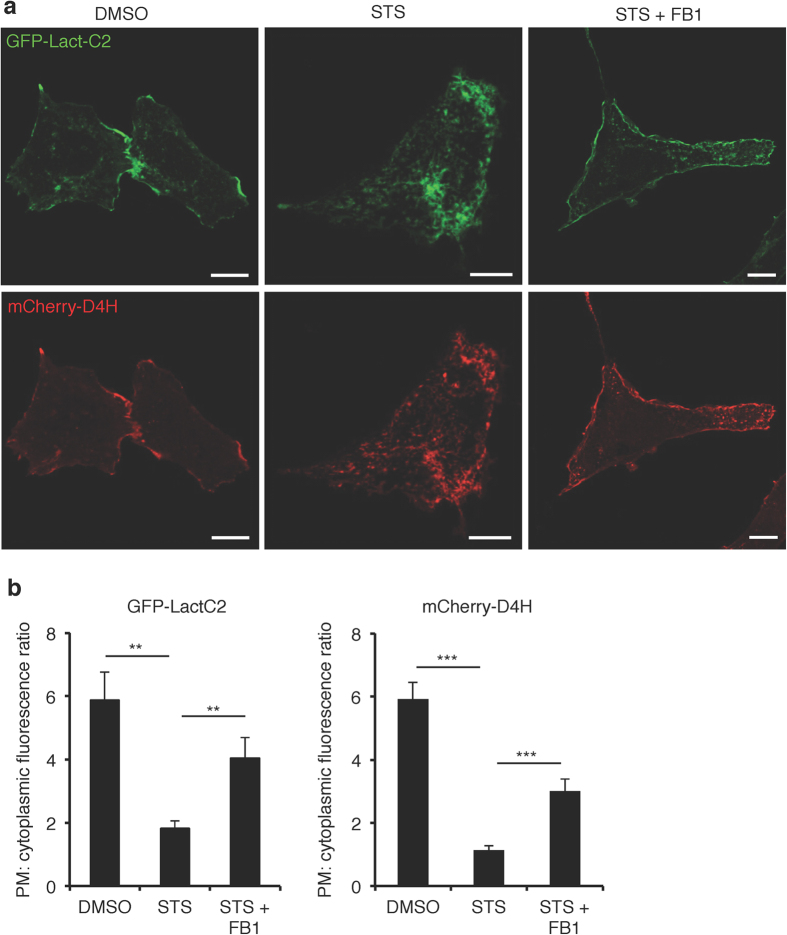
Inhibition of SM synthesis by fumonisin B1 prevents the relocalization of PtdSer. (**a**) Confocal images of CHO cells treated with DMSO, 50 nM STS or 50 nM STS + 15 μM FB1 for 24 hr expressing GFP-Lact-C2 and mCherry-D4H. Bar, 10 μm. (**b**) Quantitation of fluorescence signal plasma membrane:cytoplasm of GFP-Lact-C2 and mCherry-D4H in (**a**). Total 50 cells from three independent experiments were analysed. Data are means ± SEM. **p < 0.01; ***p < 0.001.

The loss of ORMDL proteins impairs feedback inhibition and increases SM levels. The correlation of increased SM and the mislocalization of plasmalemmal PtdSer motivated us to examine this finding in more detail. For this purpose, we observed the intracellular distribution of PtdSer in STS-CHO cells treated with the ceramide synthase inhibitor, fumonisin B1 (FB1)^33^. We determined that 15 μM FB1 would restore the levels of the major SM species to normal in STS-CHO cells (Fig. 4). We next assessed whether partial inhibition of SM biosynthetic pathway could mitigate the effects of staurosporines on the distribution of PtdSer. Consistent with the mass spectrometry determinations, the treatment of STS-CHO cells with FB1 also restored to normal the binding of GFP-NT-Lys protein to the exofacial leaflets of the PM and internal membranes (Fig. 2a–c). Simultaneous treatment of CHO cells with STS and FB1 largely prevented the redistribution of PtdSer as indicated by retention of GFP-Lact-C2 in the plasma membrane (Fig. 7a,b). Additionally, localization of the cholesterol sensor, mCherry-D4H, to the cytosolic leaflet of the plasma membrane was also restored (Fig. 7a,b).

### PtdSer mislocalization is a general feature of increased sphingomyelin content

**Figure 8:**
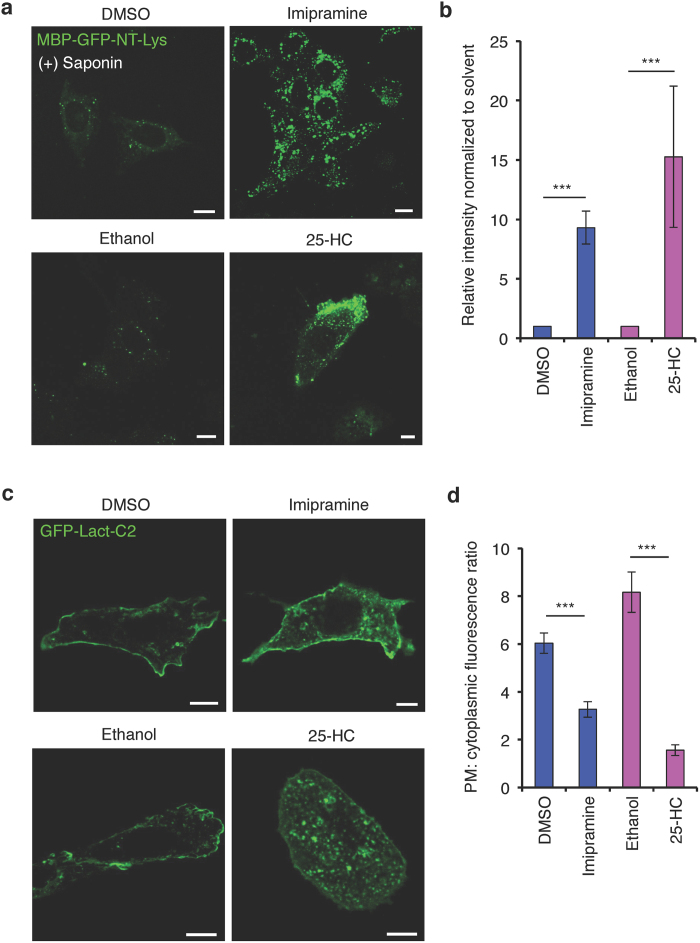
Increased SM causes redistribution of PtdSer from the plasma membrane. (**a**) Confocal images of solvent controls (DMSO and ethanol), 25 μM imipramine, and 5 μg/ml 25-HC treated CHO cells for 16 hr stained with MBP-GFP-NT-Lys protein after membrane permeabilization by 0.05% (w/v) saponin. Bar, 10 μm. (**b**) The value of fluorescence intensity of MBP-GFP-NT-Lys normalized to DMSO or ethanol in (**a**) Data are means ± SEM (50 cells from three independent experiments). ***p < 0.001. (**c**) Confocal images of CHO cells treated with DMSO, imipramine, ethanol or 25-HC expressing GFP-Lact-C2. Bar, 10 μm. (**d**) Quantitation of fluorescence signal plasma membrane:cytoplasm of GFP-Lact-C2 in (**c**). Total 50 cells from three independent experiments were analyzed. Data are means ± SEM. ***p < 0.001.

To determine if the alterations in PtdSer distribution is a general feature of excess cellular SM we sought to examine other conditions that leads to increased SM. Importantly, we choose two unrelated treatments that enhance SM by alternative mechanisms namely enhanced synthesis and inhibition of catabolism. In this regard, CHO cells were treated with imipramine, an acid SMase inhibitor^34^, or 25-hydroxycholesterol (25-HC), an oxysterol known to stimulate SM synthesis^29^,^35^. For this experiment, CHO cells were incubated with imipramine or 25-HC for 16 h, fixed, permeabilized and stained with GFP-NT-Lys to monitor the increase in cellular SM. Consistent with previous findings both treatments resulted in an increase in SM (Fig. 8a,b). Importantly, the enhancement in SM abundance was accompanied by the depletion of the GFP-Lact-C2 from the plasma membrane (Fig. 8c,d). Taken together, both the stimulation of SM biosynthesis (by STS and 25-HC) and inhibition its degradation (by imipramine) caused loss of plasmalemmal PtdSer.

## Discussion

**Figure 9:**
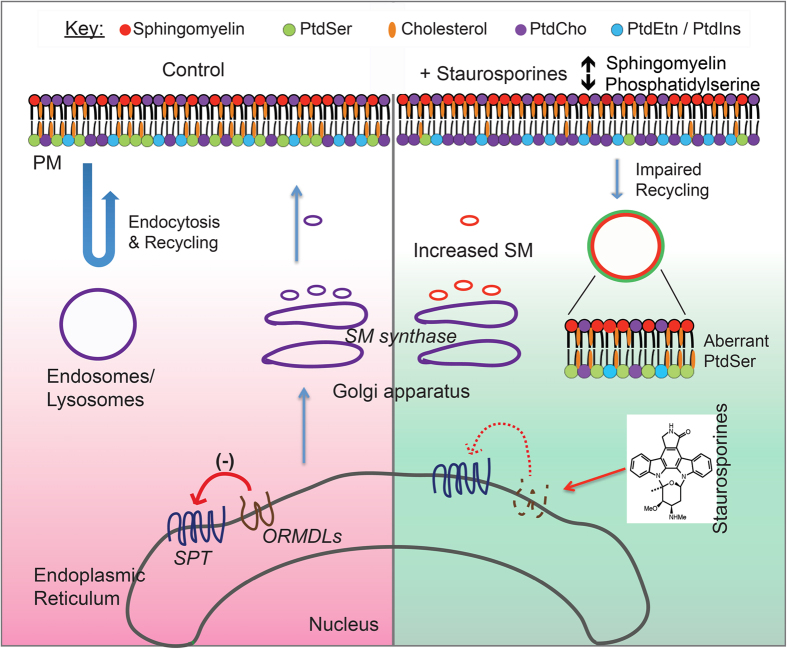
Excess Sphingomyelin impairs the recycling of PtdSer and Cholesterol out of the endolysosomal network. Under normal conditions, ORMDLs sense ceramide and act as a feedback inhibitor of the SPT complex. Levels of the plasmalemmal lipids, PtdSer, cholesterol and sphingomyelin are maintained in their relative abundance. Treatment with low-dose staurosporine results in the loss of ORMDL proteins. This in turn leads to increased levels of both plasmalemmal and endosomal sphingomyelin. Ultimately, this results in a depletion of PtdSer from the inner leaflet of the PM and accumulation in endosomal structures.

Here, we demonstrated that treatment of CHO cells with a sublethal dose of staurosporine, related compounds, 25-HC or imipramine caused the redistribution of PtdSer from the plasma membrane to endomembranes due to increased levels of SM (Fig. 9). Additionally, we found that STS and its analogs caused an increase in both plasmalemmal and endomembrane pools of SM. Metabolic tracer experiments demonstrated the increase in SM is due to enhanced biosynthesis. While STS is typically used to stimulate apoptosis, it is important to note that cells treated with 50 nM STS for 24 hr did not undergo apoptosis or expose PtdSer to the extracellular milieu^20^. Importantly, ceramide content was not changed by treatment of CHO cells with STS or OSS (Supplementary Figure 1). One possibility is that other metabolites of this pathway such as sphingosine and sphingosine 1-phosphate (S1P) could be impacted and thereby alter PtdSer distribution. However, as both of these products are the result of SM catabolism this possibility seems unlikely. Additionally, CHO cells do not express S1P receptors^36^, making it unclear how S1P would impact the cellular distribution of PtdSer. To rule out this possibility, we increased the levels of SM by treating cells with an inhibitor of sphingomyelinase. Similar to the treatments that stimulate SM synthesis, inhibiting its breakdown also caused a loss of plasmalemmal PtdSer. Taken together, we believe that increased SM is the cause of mislocalization of PtdSer in staurosporine-treated CHO cells. Intriguingly, the results demonstrate that the organization of a prototypical eukaryotic plasma membrane requires that SM levels be maintained below a threshold level, above which PtdSer relocalized to internal membranes. This finding is significant as it provides a mechanism by which the generation and maintenance of the two asymmetric leaflets of the plasma membrane is coordinated.

One question that remains is how the accumulation of an exofacial leaflet lipid, in this case, SM, impairs the trafficking of another lipid in the opposite leaflet? We have found that removal of cholesterol reduces the abundance of plasmalemmal PtdSer by ≈50% (manuscript in preparation). Thus, a simple explanation is that increased levels of SM sequesters cholesterol in the exofacial leaflet and thus stimulates the internalization of PtdSer. Additionally, high levels of lysosomal sphingomyelin caused by impaired acid sphingomyelinase activity that is seen in Niemann-Pick disease type A and B patient cells results in accumulation of PtdSer in lysosomes. To our knowledge, these patient-derived cells have normal endocytic and recycling pathways. However, Niemann-Pick type A cells have an autophagy defect, demonstrating that excess SM can impair aspects of membrane trafficking^37^. Interestingly, experiments investigating the impact of SM deficiency on vesicular trafficking in a mouse lymphoma cell line showed that sphingomyelin synthase 1 is essential for both clathrin-dependent uptake and recycling of transferrin^38^. Together, these observations suggest that SM homeostasis is critical for the normal function of the endosomal recycling pathway(s). Thus, alterations in SM metabolism may have secondary impacts on vesicular trafficking and PtdSer and cholesterol distribution. To better understand the normal PtdSer-recycling pathway, the effector(s) sensitive to changes in SM content will need to be identified.

Cellular sphingomyelin synthesis is complex and relies on a number of biosynthetic enzymes and accessory proteins. Importantly, the rate-limiting step in *de novo* SM biosynthesis is catalyzed by the serine-palmitoyltransferase complex^31^. Initially, the complex was thought to be comprised of simple heterodimers of SPT long chain base (SPTLC) subunit 1 with either SPTLC2 or 3^30^,^39^. However, recent experiments suggest that the SPT complex consists of all three SPTLC subunits^40^,^41^,^42^. The SPT complex itself does not directly sense the product of the reaction. However, the enzyme is subject to feedback inhibition mediated by the ceramide-sensing ORMDL protein family^21^,^32^,^43^. Several polymorphisms and gene expression changes have identified ORMDL gene mutations that correlate with childhood asthma^44^. Thus far, we have been unable to determine the mechanism by which STS decreases the levels of ORMDL proteins. However, our findings are congruent with previous results demonstrating that loss of ORMDL proteins leads to enhanced SPT activity and increases in SM content^21^,^32^,^43^. Recently, it was shown that addition of exogenous cholesterol induces an autophagic response that causes a reduction in ORMDL proteins^45^. However, we find that treatment of cells with low-dose STS had no impact on the subcellular localization of GFP-tagged ORMDL3 (not depicted). While another study has shown that increased SPT activity can induce upregulation of ORMDL proteins^46^. However, this mechanism does not explain our present data because treatment of CHO cells with STS resulted in decreased ORMDL proteins. In the future, determining how STS exerts it effects on ORMDLs could reveal an unappreciated mechanism of sphingolipid synthesis regulation.

Recently, de Solar *et al*. reported that sphingolipid mass increases during STS-induced apoptosis, especially in cancer cell lines^47^. In this study, treatment of human colorectal carcinoma cells with a comparatively high concentration of STS (300 nM) increased the mass of not only sphingomyelin but also dihydroceramide and ceramide^47^. Under these conditions, an increase in the expression of the ceramide synthases and a decrease in sphingomyelinase activity is seen. One difference between the previous study and our investigation is that we detected increased SM mass by in cells treated with a sublethal concentration of STS (50 nM) and devoid of the confounding metabolism that may arise from the pro-apoptotic signaling. Additionally, we did not observe ceramide accumulation in the CHO cells consistent with experiments using MDCK cells^48^. We suspect that cancer cells may also have a reduction of the ceramide-sensing ORMDL proteins to support enhanced ceramide and sphingolipid synthesis. Clearly, further investigations will be required to understand the precise regulation of ceramide and sphingomyelin synthesis and their roles in normal and malignant cells.

## Materials and Methods

### Plasmids

The pET28b vector containing the GFP-D4 was a kind gift from Dr. Yoshiko Ohno-Iwashita (Iwaki Meisei University, Japan). GFP-tagged DNA fragment containing the amino acids of 161-297 of Lysenin (GFP-NT-Lys) was amplified with the pQE30-GFP-NT-Lys vector (a kind gift from Dr. Toshihide Kobayashi, RIKEN, Japan) as a template for PCR using the following pairs of primers: 5′-GCGGGATCCGTGAGCAAGGGCGAGGAGCT-3′ (GFP sense primer) and 5′-CGCCTGCAGTTAACCAACCACTTCCAAAA-3′ (Lysenin antisense primer). The PCR product was introduced into the pMAL-C2 vector at BamHI/PstI site. The mCherry-D4H plasmid was generated as described previously^11^. The GFP-Lact-C2 is a kind gift from Dr. Sergio Grinstein (The Hospital for Sick Children, Canada).

### Purification of MBP-GFP-NT-Lys and His-GFP-D4

*E. coli.* strain BL21 (Rosetta) was used for the overexpression of MBP-tagged GFP-NT-Lys fusion proteins. *E. coli* transformed with pMAL-C2-GFP-NT-Lys were cultured in LB media at 37 °C with constant shaking until the OD600 reached 0.5. Cultures were induced with 1 mM IPTG for 5 hours at 25 °C. Next, cells were collected by centrifugation and lysed using B-PER (Pierce Biotechnology) according to the manufacturer’s instructions. Cell lysate supernatants were bound to Amylose Resin (New England BioLabs). The resin was washed with PBS (pH 7.4) and the protein was eluted with 10 mM maltose in PBS (pH 7.4). Purification was confirmed by SDS-PAGE and Coomassie staining. His-GFP-D4 was purified as previously described^11^.

### Cell culture and transfection

Wild-type CHO-K1 cells were routinely maintained at 37 °C with 5% CO_2_ in Ham’s F-12 medium (Wisent) supplemented with 5% FBS (Wisent). CHO-K1 cells were treated with 50 nM staurosporine (BioShop, Burlington, Ontario), 50 nM 7-oxostaurosporine (Santa Cruz Biotechnology), 100 nM UCN-2 (Santa Cruz Biotechnology) or 15 μM fumonisin B1 (Sigma) in Ham’s F-12 medium with 5% FBS at 37 °C for 24 hr. To determine if other treatments impact PtdSer distribution, CHO-K1 cells were treated with 25 μM imipramine (Sigma) and 5 μg/ml 25-HC (Steraloids, Rhode Island, USA) for 16 hr Ham’s F-12 medium with 5% FBS at 37 °C. For supplementation of 1-stearoyl-2-oleoyl phosphatidylserine (Avanti Polar Lipids), cells were incubated with 30 μM large multilamellar vesicles (MLVs) in serum containing Ham’s F-12 medium for 2 hr at 37 °C. CHO-K1 cells were transiently transfected with plasmids using Fugene 6 (Promega) according to the manufacturer’s instructions. Cells were fixed with 3.7% formaldehyde (FA)-PBS for 30 min at RT at 24 hr post-transfection.

### Quantitative RT-PCR

To quantify ORMDL1, ORMDL2 and ORMDL3 expression levels, first, equal amounts of cDNA were synthesized using the iScript cDNA Synthesis Kit (Bio-Rad Laboratories, Inc. Hercules, CA). Next, equal amounts of cDNA were mixed with KAPASYBR FAST qPCR Master Mix (KAPA Biosystems, Inc. Wilmington, MA). The primer sequences are as follows: ORMDL1: Forward (5′-ACATTCATGTTTGCTCGCCA-3′), Reverse (5′-TGAGGACGTACTGTCGCTGT-3′); ORMDL2: Forward (5′-ATGATGAAATGCGCAGCGTC-3′), Reverse (5′-TGACCAAGGAAAGGCTCGAC-3′); ORMDL3: Forward (5′-GGGGTGGAGAGACAGTCAAG-3′), Reverse (5′-GATGGCCAGCACATACGAGA-3′); GAPDH: Forward (5′-GACCACAGTCCATGCCATCA-3′), Reverse (5′-ATGCCGGTTAGTTTCCCGTT-3′). All qPCR was conducted at 95 °C for 3 min, and then 40 cycles of 95 °C for 1 s and 60 °C for 20 s. The specificity of the reaction was verified by melt curve analysis. The threshold crossing value was noted for each transcript and normalized to the internal control. The relative quantitation of each mRNA was performed using the comparative Ct method. Experiments were performed using a ViiA 7 system (Life technology), and data processing were performed using ViiA7 software v1.2.4.

### Western Blotting

Cell lysates were collected from a 6-well plate at each time point post STS treatment (0, 4, 8, 24 hr). Cell lysates were subjected to SDS-PAGE and immunoblotting. Antibodies used in this study include a polyclonal anti-ORMDL3 antibody (AP10739c, Abgent) raised against amino acids 53–81 and predicted to recognize all three ORMDL proteins, and an anti-GAPDH (MAB374, EMD Millipore) at a dilution of 1:1000 and 1:5000, respectively. Secondary antibodies used were anti-rabbit IgG-HRP–linked antibody (7074, Cell Signaling Technology) and anti-mouse IgG-HRP–linked antibody (7076, Cell Signaling Technology) at a dilution of 1:5000. Images were captured without the use of film by using the BIO-RAD ChemiDoc^TM^ Touch Imaging System.

### Confocal microscopy

Images were acquired using a Zeiss LSM 700 inverted confocal microscopy (Zeiss) using a Plan-Apochromat 60x/1.4 NA oil objective and acquired using Zen 2010 software (Zeiss). For any given experiment, the same scan speed and laser power were used to ensure an accurate comparison between cells and conditions. Quantisation of fluorescence intensity and correcting the brightness and contrast of the images was performed with ImageJ software (NIH) or Zen 2010 software (Zeiss). Quantification of the plasmalemmal: cytoplasm fluorescence for the Lact-C2 and D4H probes was performed using the region of interest tool in ImageJ. First, mean pixel intensities for the plasma membrane and cytoplasm were calculated for each cell. Next, the quotient of these values was obtained to determine an enrichment of probe in the PM. For display panels, post-acquisition adjustments were made homogenously across the entire image, and the linearity of mapped pixel values was not altered.

### Binding of His-GFP-D4 and MBP-GFP-NT-Lys to the exofacial leaflet of the plasma membrane

Live cells were incubated with His-GFP-D4 (15 μg/ml) or MBP-GFP-NT-Lys (20 μg/ml) in serum-free RPMI medium for 15 min at RT, washed with PBS and imaged using confocal microscopy. For quantitative analysis with a flow cytometry, cells were detached from plates with 0.05% trypsin-EDTA for 1 min at 37 °C, collected and resuspended in 0.3 ml of ice-cold PBS. Cells were analyzed using FACS Calibur (BD Bioscience) with CellQuest software (BD Bioscience).

### Labelling of intracellular sphingomyelin with MBP-GFP-NT-Lys

Cells were fixed with 3.7% FA-PBS for 30 min at RT, then permeabilized with 0.05% saponin-PBS for 10 min at RT, incubated with MBP-GFP-NT-Lys (20 μg/ml) in PBS for 15 min at RT. Cells were observed using a confocal microscopy.

### Filipin staining

Filipin specifically binds to non-esterified cholesterol^49^. Cells were fixed with 3.7% FA-PBS for 30 min at RT and then incubated with 0.5 mg/ml filipin (Polysciences, Warrington, PA) in PBS for 16 hr at 4 °C. Samples stained with filipin were visualized using a 405 nM laser of a confocal microscopy.

### Lipid analysis

Cellular unesterified cholesterol was quantified using the Amplex Red Cholesterol Assay Kit (Invitrogen), according to the manufacturer’s instructions. For lipidomics analysis of sphingomyelin and ceramide, mass spectrometry (LC/MS/MS) was used in the AFBM Mass Spectrometry Lab at the Hospital for Sick Children, Toronto, Ontario, Canada^50^.

### [^3^H]-serine labeling

Incorporation of exogenous [^3^H]serine into sphingolipids and phospholipids were determined as described previously (Ridgway 1995 JLR). Briefly, CHO-K1 cells treated with STS were incubated in serine-free medium for 3 h followed by addition of 5 μCi/ml of [^3^H]serine for 2 h. Cells were subsequently harvested, and total lipids were extracted and resolved by thin-layer chromatography. Following staining with iodine, bands corresponding to PtdSer, PtdEtn, SM and ceramide were identified based on authentic standards, scraped into vials and the radioactivity quantified by liquid scintillation counting. Total cellular protein was measured by the Lowry method.

### Statistical analysis

Statistical analysis was carried out using Student’s two-tailed *t*-test.

## Additional Information

**How to cite this article**: Maekawa, M. *et al*. Staurosporines decrease ORMDL proteins and enhance sphingomyelin synthesis resulting in depletion of plasmalemmal phosphatidylserine. *Sci. Rep.* **6**, 35762; doi: 10.1038/srep35762 (2016).

**Publisher’s note**: Springer Nature remains neutral with regard to jurisdictional claims in published maps and institutional affiliations.

## Supplementary Material

Supplementary Information
