# Supplementary material for: Staurosporines decrease ORMDL proteins and enhance sphingomyelin synthesis resulting in depletion of plasmalemmal phosphatidylserine
Source: Sci Rep. 2016 Nov 2;6:35762. doi: 10.1038/srep35762 (PMC5090970; doi:10.1038/srep35762)
Supplement: Supplementary Information [file srep35762-s1.pdf]

Supplementary Information

Staurosporines decrease ORMDL proteins and enhance sphingomyelin synthesis resulting in depletion of plasmalemmal phosphatidylserine.

Masashi Maekawa[1,#], Minhyoung Lee[1], Kuiru Wei[1], Neale D. Ridgway[2] and Gregory D. Fairn[1,3,4*]

[1]Keenan Research Centre for Biomedical Science, St. Michael's Hospital, Toronto, ON, Canada.
[2]Departments of Pediatrics, and Biochemistry & Molecular Biology, Atlantic Research Centre, Dalhousie University, Halifax, Nova Scotia, Canada
Departments of [3]Biochemistry and [4]Surgery, University of Toronto, Toronto, ON, Canada.
[#]Current address; Department of Biochemistry and Molecular Genetics, Ehime University Graduate School of Medicine, Division of Cell Growth and Tumor Regulation, Proteo-Science Center, Ehime University, Toon, Ehime, Japan.

*Author for correspondence (fairng@smh.ca)

Supplementary Figure 1

a

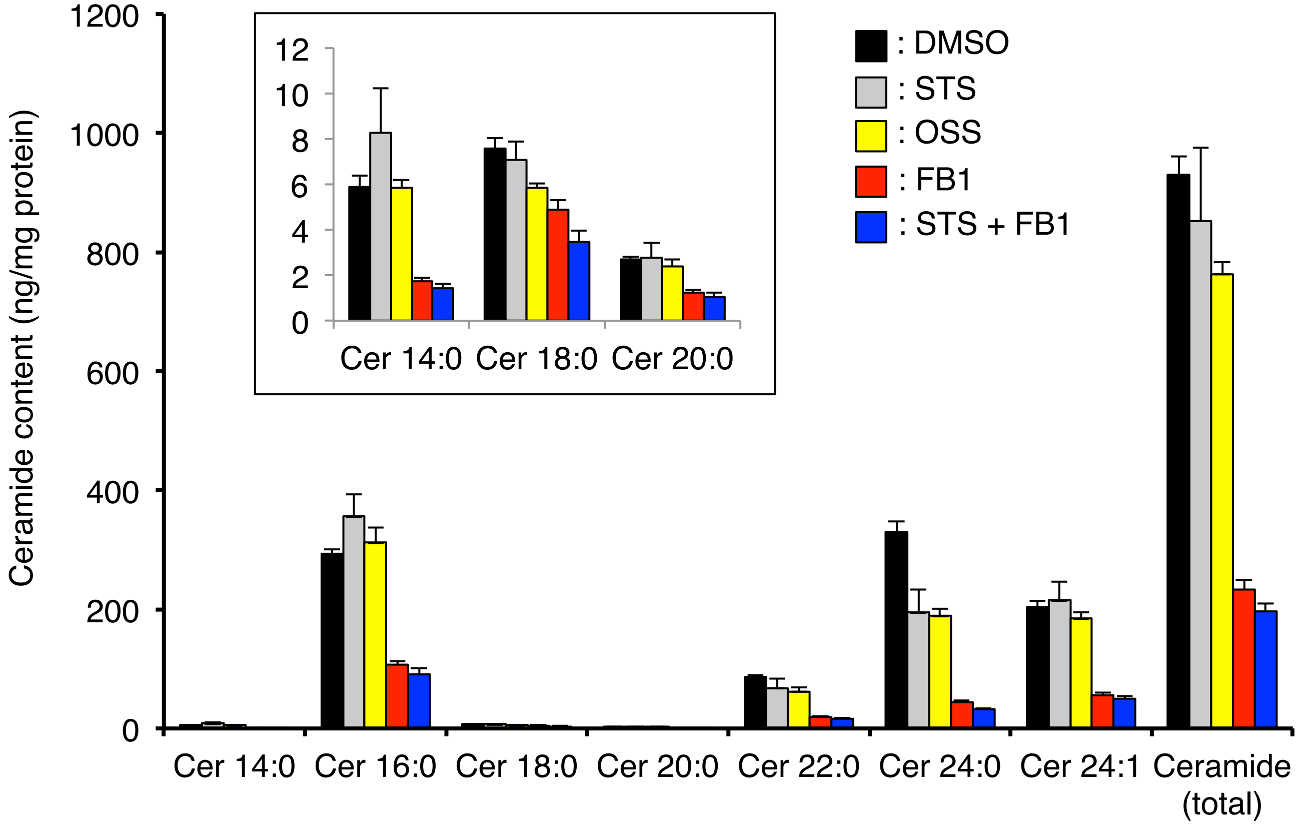

b

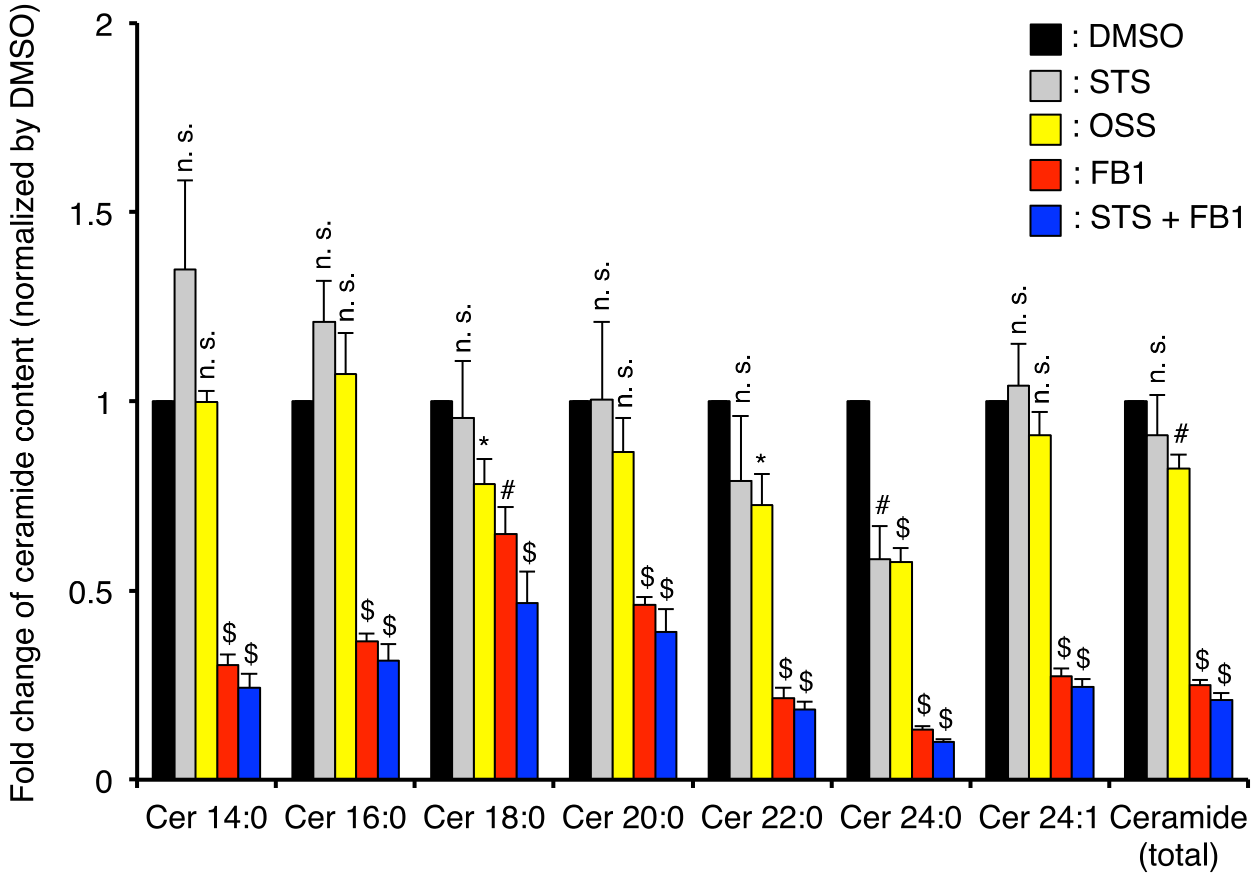

Supplementary Figure Legends

Supplementary Figure 1. Determination of the molecular species of ceramide following low-dose staurosporine. Quantitative lipidomic analysis of ceramide species from control CHO cells and treated with 50 nM staurosporine (STS), 50 nM 7-oxostaurosporine (OSS), 15 μM fumonisin B1 (FB1) and 50 nM STS + 15 μM FB1 for 24 hours. Lipid composition is shown normalized to protein to compare the changes due to incubation with the drugs (a) and then data are normalized by the value of DMSO samples (b). Data are mean values ± sem (n=4). *, $p<0.05$; #, $p<0.01$; $, $p<0.001$; n. s, not significant vs. DMSO.

Figure 6b, top portion of panel

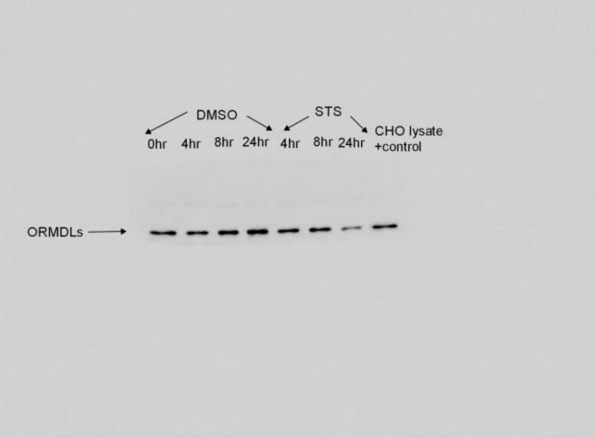

Figure 6b, bottom portion of panel

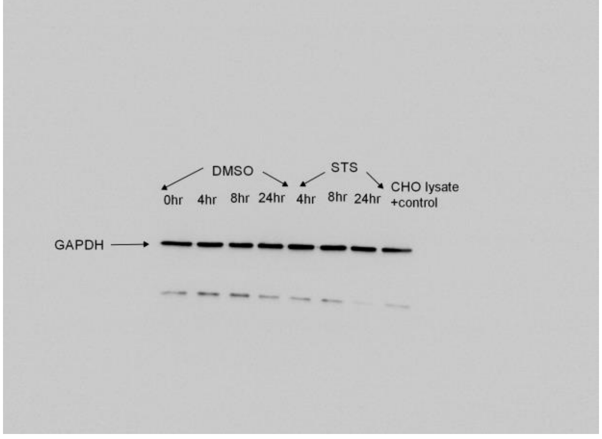

Supplementary Figure 2. Images of uncropped blots. Images of full blots that were cropped for presentation are shown. Images were acquired using the BIO-RAD ChemiDoc™ Touch Imaging System.
